# The Discovery of Zoonotic Protozoans in Fleas Parasitizing on Pets as a Potential Infection Threat

**DOI:** 10.2478/s11686-020-00221-2

**Published:** 2020-05-28

**Authors:** Olga Pawełczyk, Marek Asman, Krzysztof Solarz

**Affiliations:** Department of Parasitology, Faculty of Pharmaceutical Sciences in Sosnowiec, Medical University of Silesia, Katowice, Poland

**Keywords:** *Babesia microti*, *Toxoplasma gondii*, *Ctenocephalides felis felis*, *Ctenocephalides canis*, PCR, Nested PCR

## Abstract

**Purpose:**

Fleas are insects with a high medical and veterinary importance. They may participate in spreading of many pathogenic agents, but still there is limited information about their possible reservoir or vector role for protozoans. The main aim of this study was an attempt of detection zoonotic pathogens, such as *Babesia microti* and *Toxoplasma gondii* in fleas *Ctenocephalides felis felis* and *Ctenocephalides canis.*

**Methods:**

In 2013–2017, 155 fleas were captured from domestic dogs and cats in veterinary clinics, animal shelters and pet grooming salons in Upper Silesia Region in Poland. Then, the DNA was extracted from each *Ctenocephalides* flea by using the ammonia method. Samples were screened for the presence of *B. microti* and *T. gondii* using PCR and nested PCR methods.

**Results:**

*B. microti* was reported in 6.6% of *C. felis felis* and 9.1% of *C. canis*, whereas the prevalence of coinfection with *B. microti* and *T. gondii* was 1.9% in cat fleas and 2.3% in dog fleas.

**Conclusion:**

This study shows the first cases of *B. microti* occurrence and *B. microti* and *T. gondii* coinfection in *Ctenocephalides* fleas. The estimation of prevalence of examined protozoans may be useful considering the possibility of infection among companion animals, as well as during presentation of the potential risk of infection in humans. In order to clarify the role of *C. felis felis* and *C. canis* in transmission of *B. microti* and *T. gondii*, the another studies with in vitro cultures and laboratory animals are needed.

## Introduction

Fleas are insects with a high medical and veterinary significance. Their bites usually cause an irritation and allergic reactions. Furthermore, they may transmit many pathogenic agents with zoonotic capacity, like *Yersinia pestis*, *Francisella tularensis*, as well as species from the *Rickettsia* and *Bartonella* types [1, 2]. Fleas developed many ways of pathogen transportation, like blood-sucking or by infected excrements, by vertical, horizontal and mechanical transmission [3–5]. The species examined in this study, cat flea—*Ctenocephalides felis felis* (Bouché, 1835) and dog flea—*Ctenocephalides canis* (Curtis, 1826) are among the most frequently occurring external parasites affecting companion animals in the world [6].

According to our knowledge, epidemiological studies about flea prevalence on pets have been limited in Poland [7, 8]. The occurrence of fleas in populations of domestic animals and the prevalence of zoonotic species in examined specimens are occasionally described in foreign reports [9–11]. Moreover, there is no information about the detection or possibility of *Babesia microti* and *Toxoplasma gondii* transmission by fleas.

*B. microti*, a protozoan, which parasitizes in erythrocytes of an intermediate host, is absorbed by ticks with blood during feeding [12–14]. Thus, babesiosis is induced by the transmission of protozoans with tick saliva to organism and by transplantation or blood transfusion from infected patient [15, 16]. The first report about *B. microti* detection in Poland was confirmed in 1997 by Humiczewska et al. [17]. Nowadays, this parasite is abundant in the USA, in Eastern Europe, as well as in Poland [18–20]. To the main vectors of this pathogen belong the representatives of the Ixodina suborder, especially two of them, namely *Ixodes scapularis* and *I. ricinus* [21, 22]. The occurrence of this protozoan has been also shown in *Dermacentor reticulatus* [23, 24].

In this study, the second detected, cosmopolitan parasite was *T. gondii*, an obligatory intracellular Apicomplexa protozoan, which does not require arthropod vectors to finish its life cycle. Its prevalence ranks on high to moderate level in the natural habitat [25]. *T. gondii* undergoes the life cycle in two organisms—the final hosts of this protozoan are representatives of the Felidae family, whereas the role of intermediate hosts may fulfill both birds and mammals (including humans). The alimentary way is the most often reason of infection with *T. gondii*. The bradyzoites and tachyzoites occur in a raw meat, as well as in the other food products, whereas the *T. gondii* sporocysts may be present in potable water [25–27]. In recent years the concern of parasitologists about the *T. gondii* transmission has been increased, what is probably caused by detection of this species in ticks [28].

The present work was aimed at providing evidence of the identity of fleas on domestic cats and dogs in Poland and showing the presence of zoonotic protozoans, like *B. microti* and *T. gondii* in the examined material.

## Materials and Methods

### Collection of Material

Fleas have been collected from January 2013 to April 2017 from companion animals during the routine examination in veterinary clinics and animal shelters, as well as the beauty treatments in pet grooming salons in the cities of Upper Silesia Region in Poland, Central Europe. They were captured from domestic cats and dogs, directly from their skin or pelage. The material was conserved in plastic tubes with 70% ethyl alcohol. Fleas were determined to species and sex using stereoscopic microscope SZ-40 (Olympus, Japan), according to Skuratowicz key (1967) [29].

### DNA Extraction and Molecular Detection of Pathogens

DNA was isolated from a single individual by using the ammonia method [30]. Fleas were placed in separate sterile plastic tubes with 100 μl of 0.7 M NH_4_OH. Subsequently, insects were mechanically crushed by the homogenate CAT X 120 (Ingenieurbüro CAT, M. Zipperer GmbH, Staufen, Germany) and the samples were boiled at 100 °C for 15 min in a heating block TB-941U (JW Electronic, Warsaw, Poland). Then, lids were opened and the samples were boiled at 100 °C for 10 min in order to remove the ammonia, centrifuged for 5 min at 12 000 rpm and the supernatant was transferred to a new plastic tube. The DNA concentration was measured using the Nanospectrophotometer Pearl (Implen, Munich, Germany).

The DNA samples were screened for the presence of pathogens using PCR and nested PCR methods. The amplification reactions were conducted in a thermal cycler MJ Mini (BioRad, California, USA). To the detection of *T. gondii* in fleas collected in 2013–2014 a commercial kit PK 40 (Blirt-DNA, Gdańsk) was used, which consisted of two mixes (PCR-OUT and PCR-IN). Each mix included one pair of primers, dNTP’s, DNA TaqNova polymerase and reaction buffer. The detection consisted of the amplification of the gene fragment which coding the antigen protein 65 kDa *T. gondii*. The material was analyzed according to the manufacturer instruction. The conditions of both reactions were as follows: preliminary denaturation in 94 °C for 2 min, then denaturation in 94 °C for 30 s, annealing at 64 °C for 60 s, elongation at 72 °C for 30 s and final elongation at 72 °C for 2 min. 40 cycles in PCR reaction were performed, whereas in nested PCR – 35. In turn, the two pair of primers Pml/AS1, Pml/S1 and Pml/AS2, Pml/S2 specific to the fragment of B1 gene [31] were used for detection *T. gondii* in fleas collected in 2015–2017. Two-stage PCR reaction consisted of 30 and 20 cycles respectively was made, according to Sroka et al. [32] protocol.

*B. microti* was detected by using the two pairs of primers—Bab1/Bab4 and Bab2/Bab3 specific to the 18S rRNA gene [33]. The conditions of PCR reaction was the same as in study of Wójcik-Fatla et al. [33]. The nested PCR conditions was identical as during the first PCR reaction, but the number of reaction cycles was 30.

The PCR products were separated electrophoretically in 2% ethidium bromide-stained gels. Then the gels were visualized under ultra violet light and photographed in the analyzer Omega 10 (Ultra-Lum, California, USA). The presence of reaction products with the size of 238 bp and 154 bp for *B. microti*, 308 bp (Blirt DNA Gdańsk) or 531 bp for *T. gondii* were considered positive.

## Results

### Determination of Fleas

In total 155 fleas were collected from the same number of pets—domestic dogs (*Canis lupus familiaris*) (89) and domestic cats (*Felis catus*) (66). Four flea species were identified from the collected material. The most frequent species was *C. felis felis* (68.4%), followed by *C. canis* (28.4%), *Pulex irritans* Linnaeus, 1758 (1.9%) and *Archaeopsylla erinacei* (Bouché, 1835) (1.3%). Females constituted the majority of collected individuals (84.5%).

### Flea Species Distribution on Examined Hosts

Species diversity was greater in the material collected from domestic dogs (*C. lupus familiaris*), than from domestic cats (*F. catus*). The most numerous flea species caught from population of domestic dogs was *C. felis felis* (52.81%), then *C. canis*, *P. irritans* and *A. erinacei* (Table 1). Out of the total number of fleas (66) collected from domestic cats (*F. catus*), the vast majority constituted of cat fleas (*C. felis felis*) (89.39%). Furthermore, dog fleas (*C. canis*) were also reported (Table 1).

**Table 1:** Species and sex diversity of fleas collected from examined host species

Host species	Flea species	Sex		Total number of fleas
		Female	Male	
*Felis catus*	*Ctenocephalides felis felis*	55	4	59 (89.39%)
	*Ctenocephalides canis*	4	3	7 (10.61%)
*Canis lupus familiaris*	*Ctenocephalides felis felis*	38	9	47 (52.81%)
	*Ctenocephalides canis*	29	8	37 (41.57%)
	*Pulex irritans*	3	0	3 (3.37%)
	*Archaeopsylla erinacei*	2	0	2 (2.25%)
	Total number of fleas	131 (84.52%)	24 (15.48%)	155 (100%)

### Detection of Pathogenic Factors in Fleas

In this study, all collected cat fleas (*C. felis felis*) and dog fleas (*C. canis*) were molecularly analyzed in order to detect *B. microti* and *T. gondii.* The DNA was isolated from all *Ctenocephalides* fleas (150), including 106 individuals of *C. felis felis* (93 females and 13 males) and 44 of *C. canis* (33 females and 11 males). We excluded the DNA isolation from *P. irritans* and *A. erinacei,* which do not belong to specific flea species of companion animals. The present study shows the occurrence of two pathogenic species in cat and dog fleas removed from pets.

*B. microti* was detected both in females and males of *C. felis felis* and *C. canis*. This protozoan occurred on the similar level in both species. Its prevalence was 9.1% in *C. canis* and 6.6% in *C. felis felis* (Table 2).

**Table 2:** The number and percentage of *Ctenocephalides* fleas collected from pets, infected with *Babesia microti* and *Toxoplasma gondii*

Flea species and sex		No. of studied fleas	1 pathogen		2 pathogens
			*Babesia microti*	*Toxoplasma gondii*	*Babesia microti* + *Toxoplasma gondii*
*Ctenocephalides felis felis*	Female	93	6 (6.4%)	0 (0%)	2 (2.1%)
	Male	13	1 (7.7%)	0 (0%)	0 (0%)
Total number of *Ctenocephalides felis felis*		106	7 (6.6%)	0 (0%)	2 (1.9%)
*Ctenocephalides canis*	Female	33	2 (6.1%)	0 (0%)	1 (3%)
	Male	11	2 (18.2%)	0 (0%)	0 (0%)
Total number of *Ctenocephalides canis*		44	4 (9.1%)	0 (0%)	1 (2.3%)

In case of *T. gondii*, the single occurrence was not detected in examined material. The coinfection of *B. microti* and *T. gondii* was reported in case of 2.3% of *C. canis* and 1.9% of *C. felis felis.* Co-occurrence of these protozoans was detected only in females (Table 2).

## Discussion

In this study, new epidemiological data about the prevalence of *B. microti* and *T. gondii* in fleas collected from domestic cats and dogs were presented.

The first attempt of *B. microti* detection in fleas was made in 2013, however, the DNA of this protozoan was not reported in examined *Xenopsylla cheopis*, *C. canis*, *C. felis* and *Cediopsylla inaequalis* fleas [34]. In our study, the prevalence of this pathogen was 6.6% of *C. felis felis* and 9.1% of *C. canis. B. microti* is a species, which life cycle is adapted to the tick physiology. It belongs to organisms which are latent in improper conditions, thus the transmission of this protozoan from tick to another organism takes approximately 36 to 72 h. This process is caused by a long time of sporoblasts activation and the production of sporozoites in the tick salivary glands. The active transmission of this pathogen by cat or dog fleas is probably impossible, because of a short flea parasitizing period and inability to close the *B. microti* development cycle in the fleas body [21, 35, 36]. On the other hand, there is still lack of knowledge about the behavior of *B. microti* gamonts in the flea intestine, which would be decisive in determining the possibility of the passive transmission process.

Alimentary way of transmission does not clarify a high prevalence of *T. gondii* in many of herbivorous mammals (up to 75%) [37, 38], wild rodents (up to 35%) [39, 40] and fowl (even up to 100%) [41], which should have limited contact with the invasive forms of this protozoan. For this reason, other ways of *T. gondii* transmission are considering, including – by blood-sucking arthropods [42–44]. Deryło et al. [45] as the first showed the experimental transmission of *T. gondii* in *I. ricinus*, when they found tachyzoites and bradyzoites in tissues of nymphs and females of this tick species. The first molecularly confirmed detection of *T. gondii* in *I. ricinus* was described in 2003, what initiated interest of this topic [28]. Presence of this species was reported in ticks collected from the north-eastern region of Poland. Moreover, in that study development of infection was confirmed in  a culture of laboratory mice, as an effect of their previous inoculation by homogenate, composed of *T. gondii* infected *I. ricinus* ticks [31]. In another research, from the Upper Silesia Region in Poland, the presence of *T. gondii* was molecularly detected in questing *I. ricinus*, and also in ticks collected directly from the hosts. What is interesting, the prevalence of this protozoan in ticks, collected from dogs and cats in study of Asman et al. [46] was very high at this time and oscillated about 98%. It suggested, that pets living in the Silesia Region are appropriate hosts for *T. gondii*, what confirmed the validity of our study. In contrast, a low prevalence of *T. gondii* in *I. ricinus* collected from Shetland ponies was reported (2.99%), what may indicate, that these animals are not the competent hosts for this protozoan [47]. In other studies, the occurrence of *T. gondii* in *D. reticulatus* and *Haemaphysalis longicornis* was reported [23, 48]. Zhou et al. [48] conducted a study, which focused on showing the role of *H. longicornis* in transmission of *T. gondii*. In this experiment, the blood-feeding transmission in adult ticks was not observed, but ticks became infected when they ingest *T. gondii*, which was present in blood of mammals. Thereby, accidental ingestion of infected *H. longicornis* tick may induce a mechanism of *T. gondii* transmission between ticks and hosts. This way of transmission, could explain many cases of toxoplasmosis among herbivorous species. It is possible, because of a similar way of transmission in case of another protozoan species from the Apicomplexa type—*Hepatozoon canis* [48, 49]. In turn, Skotarczak [50] suggested, that if *T. gondii* is a species with asexual reproduction in different cells of intermediate hosts, which migrates also to monocytes and neutrophils that there is a possibility of its transmission to other hosts by other hematophagous arthropods.

Our paper presents the occurrence of *T. gondii* DNA in examined flea species. This protozoan is present only in fleas, which are also coinfected with *B. microti*. The prevalence of this co-occurrence in *C. felis felis* estimated at 1.9%, while in case of *C. canis*—2.3%. In our study, the DNA of *T. gondii* was detected only in females, what was probably caused by their larger number in both examined populations and a greater life activity correlated to the higher blood-sucking quantity, when they prepare to procreation [51].

The first cases of *B. microti* detection and coinfection of *B. microti* and *T. gondii* in *Ctenocephalides* fleas was shown in this study. The phenomenon of *B. microti* and *T. gondii* passive transmission (by swallowing an infected flea) by *C. felis felis* and *C. canis* may exist. However, it requires further research, in order to test the viability of gamonts in the flea intestine tract and the possibility of laboratory animals infection via oral route.
